# Ginseng ginsenoside pharmacology in the nervous system: involvement in the regulation of ion channels and receptors

**DOI:** 10.3389/fphys.2014.00098

**Published:** 2014-03-19

**Authors:** Seung-Yeol Nah

**Affiliations:** Ginsentology Research Laboratory, Department of Physiology, College of Veterinary Medicine and Bio/Molecular Informatics Center, Konkuk UniversitySeoul, South Korea

**Keywords:** ginseng, ginsenosides, ion channels and receptors, interaction site(s), nervous system

## Abstract

Ginseng, the root of Panax ginseng C.A. Meyer, is one of the oldest traditional medicines and is thought to be a tonic. It has been claimed that ginseng may improve vitality and health. Recent studies have advanced ginseng pharmacology and shown that ginseng has various pharmacological effects in the nervous system. Ginsenosides, steroid glycosides extracted from ginseng, were one of the first class of biologically active plant glycosides identified. The diverse pharmacological effects of ginsenosides have been investigated through the regulation of various types of ion channels and receptors in neuronal cells and heterologous expression systems. Ginsenoside Rg_3_ regulates voltage-gated ion channels such as Ca^2+^, K^+^, and Na^+^ channels, and ligand-gated ion channels such as GABA_A_, 5-HT_3_, nicotinic acetylcholine, and N-methyl-D-aspartate (NMDA) receptors through interactions with various sites including channel blocker binding sites, toxin-binding sites, channel gating regions, and allosteric channel regulator binding sites when the respective ion channels or receptors are stimulated with depolarization or ligand treatment. Treatment with ginsenoside Rg_3_ has been found to stabilize excitable cells by blocking influxes of cations such as Ca^2+^ and Na^+^, or by enhancing Cl^−^ influx. The aim of this review is to present recent findings on the pharmacological functions of the ginsenosides through the interactions with ion channels and receptors. This review will detail the pharmacological applications of ginsenosides as neuroprotective drugs that target ion channels and ligand-gated ion channels.

## Introduction

**Figure 1:**
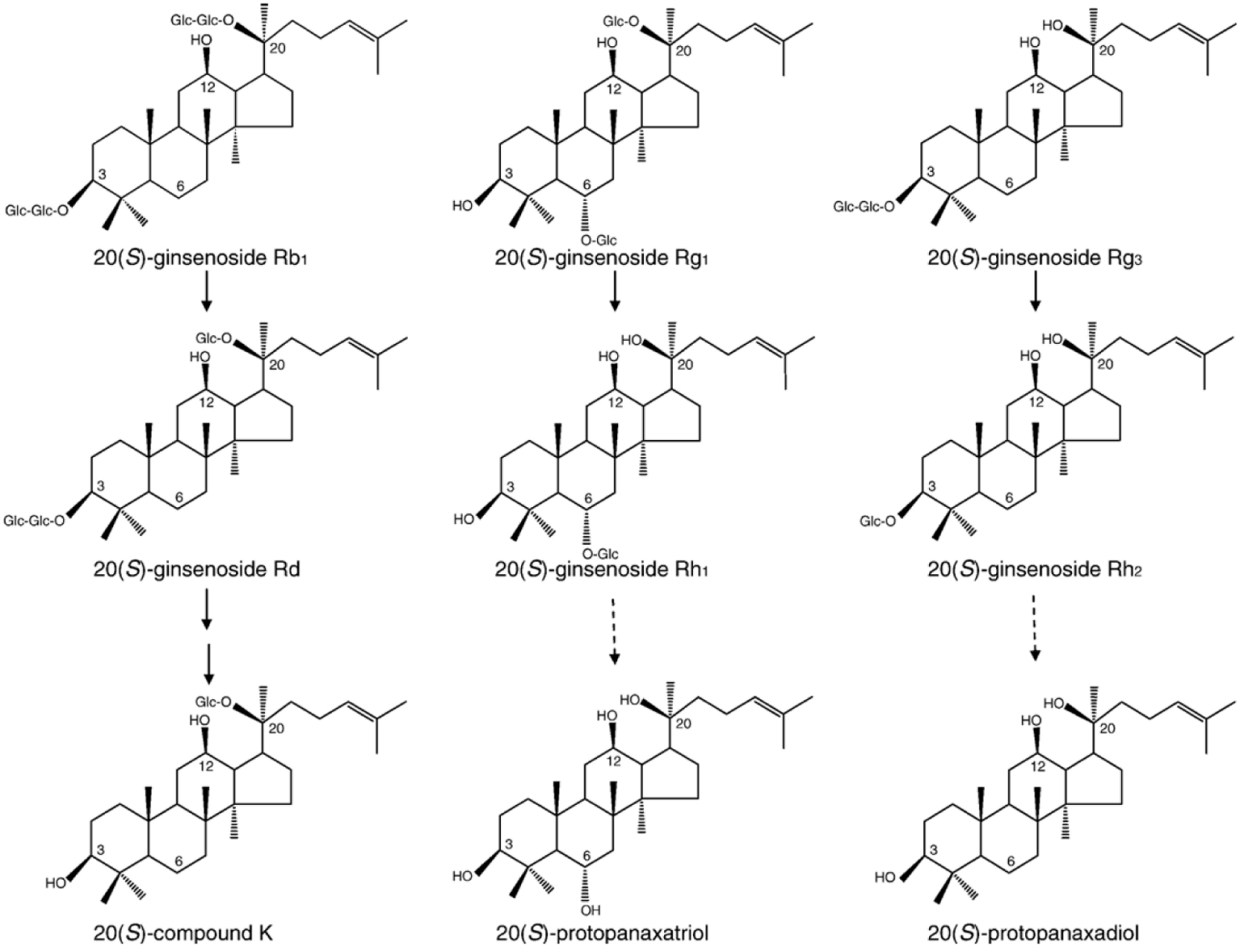
**Structures and main metabolic pathways of 20(*S*)-ginsenoside Rb1, 20(*S*)- ginsenoside Rg1, and 20(*S*)- ginsenoside Rg_3_.** Ginsenosides are known to be metabolized by human intestinal microflora. This scheme represents the structures and proposed metabolic pathways of 20(*S*)-ginsenoside Rb1, 20(*S*)-ginsenoside Rg1, and 20(*S*)-ginsenoside Rg_3_. Bold arrow: main pathway. Dotted arrow: minor or weak pathway. Adapted from Nah et al. (2007).

Ginseng, the root of *Panax ginseng* C.A. Meyer, contains a variety of ingredients useful in herbal medicines (Tyler, 1995). Ginseng glycosides, also called ginsenosides or ginseng saponin, are derivatives of triterpenoid dammarane, which consists of 30 carbon atoms. Each ginsenoside has a common hydrophobic four ring steroid-like structure with carbohydrate moieties attached (Nah et al., 2007). Several types of ginsenosides have been isolated and identified from the roots of various ginseng species from America, China, and Korea. They are mainly classified as protopanaxadiol (PD), protopanaxatriol (PT), oleanolic ginsenosides, and ginsenoside metabolites according to the position of different carbohydrate moieties at the carbon-3 and carbon-6 positions, as well as the aliphatic side chain (Figure 1). Recent studies have demonstrated that ginsenosides exhibit a variety of pharmacological effects in nervous and non-nervous systems. A line of accumulating evidence shows that ginsenoside Rg_3_, the most active ginsenoside, interacts and regulates voltage-gated ion channels and ligand-gated ion channel activity through interaction with specific amino acid(s) at channel entryways or channel pore regions that are associated with ion influx or efflux (Lee et al., 2004b). This review will describe the physiology and pharmacology of ginseng ginsenoside in the regulation of voltage-gated ion and ligand-gated ion channel activities through interactions with specific amino acids of channel proteins and receptors.

## Ginsenoside pharmacology through the interaction with voltage-gated ion channels

### Regulation of voltage-gated Ca^2+^ channels by ginsenosides

Ginseng extract and ginsenosides inhibit Ca^2+^ channel currents in sensory neurons. Among the various ginsenosides such as Rb_1_, Rc, Re, Rf, and Rg_1_, ginsenoside Rf was found to be the most effective in inhibiting Ca^2+^ channel activities (Nah et al., 1995). Ginsenosides also inhibit Ca^2+^ channels in rat chromaffin cells, which are one of the representative neurosecretory cells involved in catecholamine release under various stress conditions (Kim et al., 1998a). The ginsenosides listed in order of inhibitory potency on Ca^2+^ channels in rat chromaffin cells are: ginsenoside Rc > Re > Rf > Rg_1_ > Rb_1_. Ginsenosides showed a selectivity in Ca^2+^ channel regulation, inhibiting N-, P-, and Q/R-, but not L-type Ca^2+^ channels in bovine chromaffin cells (Choi et al., 2001). Ginsenoside Rg_3_ more potently inhibits L-, N-, and P-type Ca^2+^ channels than other ginsenosides tested in rat sensory neurons (Rhim et al., 2002). Ginsenoside Rb1 selectively inhibits L-type Ca^2+^ channel activity in cultured rat hippocampal neurons (Lin et al., 2012). Besides neuronal Ca^2+^ channels, ginsenoside Re also selectively inhibits L-type Ca^2+^ channel activity in guinea pig cardiomyocytes (Bai et al., 2003, 2004). In addition, ginsenosides attenuated the stimulation of membrane capacitance increase (ΔC_m_) in rat chromaffin cells. The ginsenosides listed in order of inhibitory potency on ΔC_m_ are: ginsenoside Rf > Rc > Re > Rg_1_ > Rb_1_ (Kim et al., 1998a). The regulation of Ca^2+^ channel activity and membrane capacitance by ginsenosides indicates that ginsenosides are closely involved in the regulation of neurotransmitter release from presynaptic nerve terminal(s) (Duan and Nicholson, 2008).

### Identification of ginsenoside interaction sites in voltage-gated Ca^2+^ channel regulation

**Figure 2:**
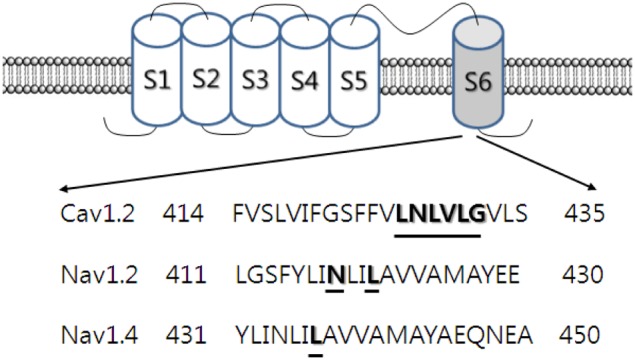
**Topology and sequence alignment of Cav1.2, Nav1.2, and Nav1.4 channels.** Ca^2+^ and Na^+^ channels consist of the main α subunit and other auxiliary subunits. α subunit has 4 domains and each domains contain 6 segments. Each segment 4 plays a membrane voltage sensor and segments 5 and 6 form channel pore. The mutated amino acid residues are underlined in the pore-forming segment 6. The underlined specific amino acid residues in these channels are thought to interact with 20(*S*)-ginsenoside Rg_3_ (Rg_3_) (Lee et al., 2008b; Choi et al., 2009).

Among the various domains of the L-type Ca^2+^ channel protein, mutations in L427R, N428R, and L431K in transmembrane domain-I-segment 6 (IS6) of the channel significantly attenuated the action of ginsenoside Rg_3_, resulting in a shift to the right in dose-response curves, although the inhibitory effects of ginsenoside Rg_3_ on Ca^2+^ channel currents was not completely abolished (Choi et al., 2009). In addition, while ginsenoside Rg_3_ treatment produced a negative shift in the inactivation voltage, it did not alter the steady-state activation voltage, and none of the mutant channels affected the ginsenoside Rg_3_-induced negative shift in inactivation voltage. Ginsenoside Rg_3_ had no effect on the inactivation time constant in wild-type and mutant channels. Thus, mutations in L427R, N428R, and L431K in transmembrane domain-I-segment 6 (IS6) of the channel partially attenuated ginsenoside Rg_3_ action. The Leu427, Asn428, and Leu431 residues of the transmembrane domain-I-segment 6 of L-type Ca^2+^ channels play important roles in the regulation of L-type Ca^2+^ channels by ginsenoside Rg_3_ (Choi et al., 2009) (Figure 2).

### Regulation of voltage-gated K^+^ channels by ginsenosides

Ginseng total saponins and ginsenoside Rg_3_ have been shown to activate Ca^2+^-activated K^+^ and ATP-sensitive K^+^ channels in rabbit coronary artery smooth muscle cells (Chung and Kim, 1999; Chung and Lee, 1999). Ginsenosides activate Ca^2+^-activated K^+^ channels in vascular smooth muscle cells (Li et al., 2001). In addition, the external application of ginseng total saponin fraction and ginsenoside Rg_3_ to rabbit coronary artery smooth muscle cells has been shown to increase the amplitude of whole-cell BK_Ca_ currents (Chung and Kim, 1999). Treatment with ginseng total extract relaxed rabbit vaginal tissue via hyperpolarization of BK_Ca_ channel activation (Kim et al., 2008a). In guinea pig cardiomyocytes, ginsenoside Re enhanced the delayed rectifier K^+^ channel (*I*_*Ks*_) (Bai et al., 2003, 2004). Ginsenoside-induced relaxation of blood vessels and other smooth muscles may be achieved via activation of K^+^ channels (Kim et al., 1999).

By contrast, ginsenoside Rg_3_ inhibits voltage-dependent Kv1.4 and Kv4.2 in human cells, but not Kv1.3, Kv1.5, and Kv2.1 expressed in *Xenopus laevis* oocytes (Jeong et al., 2004; Lee et al., 2013c), indicating that ginsenoside Rg_3_ differentially regulates Kv channel subtypes. The regulatory effect of ginsenoside Rg_3_ on Kv1.4 channel activity has been found to be strongly dependent on the extracellular K^+^ concentration, by shifting the ginsenoside Rg_3_ concentration-response curve to the right, indicating that ginsenoside Rg_3_ competes with extracellular [K^+^] for the same interaction site(s) (Lee et al., 2008a). Further study showed that the inhibitory effects of ginsenoside Rg_3_ on Kv1.4 channel currents were abolished by K^+^ activation, which is induced by increasing extracellular K^+^ concentrations. In addition, some subsets of Kv channel currents are also affected by extracellular and intracellular tetraethylammonium (TEA), which is a well-known K^+^ channel blocker. The wild-type Kv1.4 channel, however, is nearly insensitive to TEA (Pardo et al., 1992; Lee et al., 2008a). Thus, although extracellular TEA treatment did not inhibit the wild-type Kv1.4 channel, it appeared that extracellular TEA competed with ginsenoside Rg_3_ to inhibit Kv1.4 channel currents by shifting the ginsenoside Rg_3_ concentration-response curve to the right (Lee et al., 2008a). Based on these results, ginsenoside Rg_3_ may have specific interaction site(s) for Kv1.4 channel activity regulation.

### Ginsenoside Rg_3_ interacts with the extracellular tea binding site to regulate Kv1.4 channel activity

**Figure 3:**
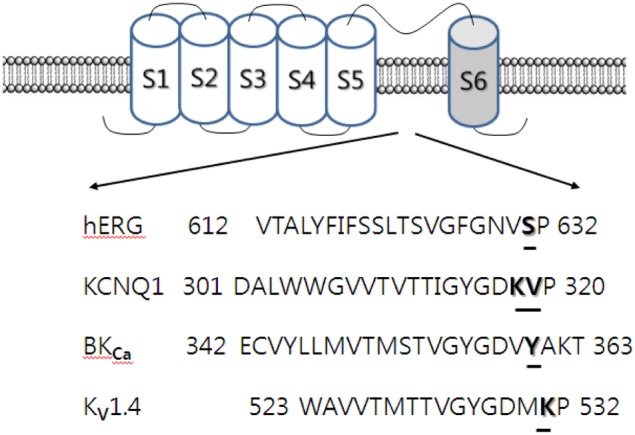
**Topology and sequence alignment of BK_Ca_, hERG, KCNQ1, and Kv1.4 K^+^ channels.** BK_Ca_, hERG, KCNQ1, and Kv1.4 K^+^ consist of the main α subunit and auxiliary β subunits. Tetramer of α subunit forms a functional K^+^ channel. α subunit contains 6 segments. Each segment 4 plays a membrane voltage sensor and segments 5 and 6 form channel pore. The mutated amino acid residues are underlined in the pore-forming segment 5 and 6. The underlined specific amino acid residues in those K^+^ channels are thought to interact with 20(*S*)-ginsenoside Rg_3_ near the K^+^ channel “signature sequence” of –TXGYGD– (Lee et al., 2008a; Choi et al., 2010, 2011a,b).

The K^+^ activation site of the Kv1.4 channel, which is located at the outer pore entry, consists of several amino acids including lysine 531 (K531) (Claydon et al., 2004). In addition, one of the extracellular TEA binding sites also contains the K531 residue. Mutations in this K531 residue to tyrosine (i.e., K531Y) increased the sensitivity of the Kv1.4 channel to extracellular TEA, abolished K^+^ activation, and also abolished the effect of ginsenoside Rg_3_ on the Kv1.4 channel. Thus, ginsenoside Rg_3_-mediated action of Kv1.4 channel activity may occur through common interaction site(s) for K^+^ activation and TEA binding sites. Alternatively, the ginsenoside Rg_3_ interaction site(s) may overlap or share the K^+^ activation site or the TEA binding site, as shown by Lee et al. (2008a) using various Kv1.4 channel mutations, including the K531 residue. Mutations have been generated in channel pore sites (S510K, D513Q, V525L, and V535Q) and outer pore sites (K531A, P532A, I533A, T534A, and V535A) (Watanabe et al., 2004). Kv1.4 channel mutations have also been generated in the N-glycosylation site (N353Q) (Judge et al., 1999), the voltage sensor site (R447C and R450C) (Claydon et al., 2004), the voltage shift sites (L478F and G548P) (Bett and Rasmusson, 2004), the pH sensitive site (H507Q), and the C-type inactivation site (V560A) (Claydon et al., 2004). The K531A mutant, located in one of the outer pores, significantly attenuated ginsenoside Rg_3_ inhibition of Kv1.4 channel currents, while the other mutations had no significant effect. Thus, ginsenoside Rg_3_ regulates Kv1.4 channel activity by interacting with Lys531, which is also known to be one of the K^+^ activation sites and one of the extracellular TEA binding sites. Other mutant channels at the K531 residue, such as K531Y, I533M, and K531Y-I533M, showed that the K531Y substitution, but not I533M, and the K531Y-I533M double substitution mostly abolished ginsenoside Rg_3_ inhibition of Kv1.4 channel currents (Lee et al., 2008a). Ginsenoside Rg_3_-mediated action of Kv1.4 channel activity is closely associated with the Lys531 residue (Figure 3).

Using homology and virtual docking model methods, which give three-dimensional configurations, Lee et al. (2008a) demonstrated that ginsenoside Rg_3_ could bind to the Kv1.4 channel protein through various interactions such as hydrogen bonds or hydrophobic interactions. Therefore, the carbohydrate portion of ginsenoside Rg_3_ plays an important role in its interaction with the Kv1.4 channel. The second, but not the first, carbohydrate attached at carbon-3 of the ginsenoside Rg_3_ backbone forms six hydrogen bonds with amino acids in the pore entryway of the Kv1.4 channel. Among the amino acids forming hydrogen bonds with ginsenoside Rg_3_, K531 forms three bonds, and threonine and histidine form the other hydrogen bonds. In addition, the ginsenoside Rg_3_ backbone is located in the pore portion of the Kv1.4 channel, enabling it to block the pore and interrupt K^+^ efflux when the channel is stimulated by depolarization. The backbone of ginsenoside Rg_3_ may act as a physical plug or wedge in ginsenoside Rg_3_-mediated Kv1.4 channel regulation. However, the K531Y mutant in the Kv1.4 channel forms only two hydrogen bonds with ginsenoside Rg_3_. Thus, the use of site-directed mutagenesis, as well as homology and virtual docking model methods, are useful tools to confirm and identify ginsenoside Rg_3_ interaction sites on the Kv1.4 channel (Figure 3).

### Ginsenoside Rg_3_ regulates BK_Ca_ channel activity through interaction with amino acid residues homologous to the Kv1.4 channel

Ginsenoside Rg_3_ enhanced BK_Ca_ channel currents, in contrast to its inhibitory effect on Kv1.4 channel currents (Choi et al., 2011a). Interestingly, BAPTA, a Ca^2+^ chelator, did not block ginsenoside Rg_3_-induced enhancement of BK_Ca_ channel currents, indicating that ginsenoside Rg_3_-enhancement of BK_Ca_ channel currents was independent of intracellular Ca^2+^. The BK_Ca_ channel was sensitive to TEA, with a significant shift in the concentration response curve of ginsenoside Rg_3_ to the right in the presence of TEA, and *vice versa* in the wild-type channel, indicating that ginsenoside Rg_3_ competes for extracellular TEA binding site(s), similarly to mutant Kv1.4 channel interaction (Lee et al., 2008a). In addition, Choi et al. (2011a) reported that ginsenoside Rg_3_-induced enhancement of the BK_Ca_ channel current was independent of the β subunit, suggesting that ginsenoside directly interacts with the BK_Ca_ channel α subunit. At the channel pore entry of the α subunit in mutant BK_Ca_ channels, the effect of ginsenoside Rg_3_ on BK_Ca_ channel current enhancements was also almost abolished, for instance, in Y360I mutant channels, in which the residue is located at near K^+^ channel signature sequence, which is also known as the extracellular TEA binding site of the BK_Ca_ channel (Choi et al., 2011a) (Figure 3).

### Ginsenoside Rg_3_ also regulates hERG K^+^ (I_*Kr*_) and KCNQ K^+^ channel (I_*Ks*_) activity through interaction with amino acid residues homologous to the Kv1.4 channel

Ginsenosides have been shown to exhibit anti-hypertension and cardio-protective effects (Attele et al., 1999). Ginseng extract shortens action potential duration, whereas ginsenoside Re has been shown to regulate the *I*_*Kr*_ and *I*_*Ks*_ channel currents of guinea pig cardiomyocytes (Bai et al., 2003; Furukawa et al., 2006). However, relatively little is known at the molecular level about how ginseng extract and ginsenosides shorten action potential duration through the activation of hERG (*I*_*Kr*_) and KCNQ (*I*_*Ks*_) K^+^ channels. Ginsenoside Rg_3_ has been shown to regulate hERG (*I*_*Kr*_) K^+^ channel by enhancing *I*_*hERG*_ and *I*_*tail*_. Ginsenoside Rg_3_ not only caused a persistent *I*_*deactivating−tail*_ without decay but also decelerated deactivating time constants. The mutation of S631 to S631C in the hERG α subunit has been reported to abolish ginsenoside Rg_3_-mediated action of hERG K^+^ channels. Thus, ginsenoside Rg_3_ enhanced *I*_*hERG*_ and *I*_*tail*_, and induced a persistent *I*_*deactivating−tail*_ with delayed deactivation of the hERG K^+^ channel through interaction with the S631 residue (Choi et al., 2011b) (Figure 3).

Ginsenoside Rg_3_ enhances *I*_*Ks*_ currents, indicating that it regulates the KCNQ (*I*_*Ks*_) K^+^ channel, which consists of KCNQ1 and KCNE1 subunits (Choi et al., 2010). Site-directed mutagenesis has shown that the K318 and V319 residues of the KCNQ1 or KCNQ1 plus KCNE1 channels are involved in ginsenoside Rg_3_-mediated action of KCNQ1 plus KCNE1 channel activity (Figure 3). Homology docking models show that the K318 residue plays an important role in the interaction of ginsenoside Rg_3_ in the closed or open state of the channels (Choi et al., 2010). Ginsenoside Rg_3_ interacts with the S631 residue of the hERG K^+^ channel and the K318 and V319 residues of the KCNQ1 plus KCNE1 channel, respectively.

### Regulation of voltage-gated Na^+^ channel by ginsenosides

Ginsenosides also regulate Na^+^ channel activities, for instance, inhibiting neuronal Na^+^ channels expressed in tsA201 cells and *Xenopus laevis* oocytes. Higher concentrations of ginseng extract and ginsenoside Rb_1_ than those used in other channel interaction experiments were required to inhibit Na^+^ channel currents (Liu et al., 2001). Ginsenoside Rg_3_ was highly potent in inhibiting Na^+^ channel currents compared to ginseng extract and the other ginsenosides tested (Jeong et al., 2004). In structure-activity relationships of the ginsenoside Rg_3_ stereoisomers, 20(*S*)-ginsenoside Rg_3_ but not 20(*R*)-ginsenoside Rg_3_ inhibited the neuronal Na^+^ channel currents in a dose- and voltage-dependent manner. The hydroxyl group at carbon-20 of 20(*S*)-ginsenoside Rg_3_ may be geometrically better aligned with the hydroxyl acceptor group in the ion channels than that of the 20(*R*)-ginsenoside Rg_3_ (Jeong et al., 2004).

A structure-activity relationship study of ginsenoside Rg_3_ investigated the role of the aliphatic side chain, [-CH_2_CH_2_CH = C(CH_3_)_2_], which is coupled to carbon-20 of the 20(*S*)-ginsenoside Rg_3_ backbone in Na^+^ channel interaction (Lee et al., 2008c). The reduction of the double bond in the aliphatic side chain of 20(*S*)-ginsenoside Rg_3_ strengthens the inhibitory effect on Na^+^ channel activity, shifting the concentration-response curve significantly to the left. However, deletion, hydroxylation, or oxygenation of the aliphatic side chain caused an attenuation or loss of Na^+^ channel current inhibition. The aliphatic side chain of 20(*S*)-ginsenoside Rg_3_, as well as the hydroxyl group of carbon-20 of ginsenoside Rg_3_ stereoisomers, plays an important role in Na^+^ channel regulation. Thus, the aliphatic side chain of ginsenoside Rg_3_ may be a future target for chemical modifications to tune regulation of Na+ channels by Rg_3_.

In further studies on ginsenoside Rg_3_-mediated voltage-gated neuronal Na^+^ channel interaction, two main characteristics have been identified. One is that ginsenoside Rg_3_ treatment causes a depolarizing shift in the activation voltage step in wild-type Na^+^ channels, indicating that ginsenoside Rg_3_ binding to the Na^+^ channel does not allow the Na^+^ channel to easily open at a given voltage step, requiring greater depolarizing stimulation compared to untreated channels (Lee et al., 2005). The other characteristic is that ginsenoside Rg_3_ induces use-dependent inhibition, meaning that the channel pore-blocking actions of ginsenoside Rg_3_ are enhanced by rapid, repeated stimulation over a very short time period, indicating that ginsenoside Rg_3_ may be a kind of open channel blocker.

The idea that ginsenoside Rg_3_ is a kind of open channel blocker of Na^+^ channel is supported by experiments using inactivation-deficient Na^+^ channel mutants, in which the inactivation gate has been mutated from IFM to Q3, and transient inward currents are converted into long-lasting inward currents (Lee et al., 2005). Ginsenoside Rg_3_ more potently inhibited the plateau than peak *I*_Na_, and facilitated channel closing in inactivation-deficient channel mutants. Interestingly, mutations in one amino acid (Lys859 to Glu859 in brain Na_*V*_1.2 channel) in the voltage-sensor domain in the S4 helix abolished the ginsenoside Rg_3_-mediated depolarizing shift without affecting the ginsenoside Rg_3_-mediated inhibition of peak current (*I*_Na_). These results indicate that ginsenoside Rg_3_ may have interaction sites for the brain Nav1.2 channel regulation.

### Identification of ginsenoside interaction sites in voltage-gated Na^+^ channel regulation

Although ginsenoside Rg_3_ regulates neuronal Na^+^ channels as a kind of open channel blocker, showing use-dependent inhibition and a depolarizing shift in the activation curve of wild-type Na^+^ channels, the exact interaction sites in the Na^+^ channel proteins have not been identified. Batrachotoxin (BTX) is a neurotoxin that acts on Na^+^ channels. BTX toxin was first found in the skin of the South American frog *Phyllobates terribilis*, and persistently activates brain Na_*V*_1.2 and skeletal muscle Na_*V*_1.4 channels, rather than inhibiting Na^+^ currents as lidocaine and tetrodotoxin do (Wang and Wang, 1998). In addition, BTX is a steroidal alkaloid toxin, with a backbone structure similar to that of ginsenoside Rg_3_. Interestingly, ginsenoside Rh_2_ inhibited [^3^H]BTX-B binding in rat brain membrane fractions and attenuated glutamate release (Duan et al., 2006; Duan and Nicholson, 2008), showing that the ginsenoside Rg_3_-induced interaction with rat brain Na_V_1.2 channel activity may involve the BTX binding sites, and that the interference by ginsenoside of [^3^H]BTX-B binding in rat brain membrane fractions is relevant to ginsenoside Rg_3_-induced Na^+^ channel regulation. BTX interaction sites are located at the I433, N434, and L437 residues of the Na_V_1.4 channel and equivalent residues such as I417, N418, and L421 of brain Na_V_1.2 channels in domain-I segment 6 (IS6).

Channel mutations in BTX binding sites, such as N418K and L421K in rat brain Na_V_1.2, and L437K in the mouse skeletal muscle Na_V_1.4 channel, have been shown to attenuate or abolish ginsenoside Rg_3_ inhibition of Na^+^ currents (Figure 2). In addition, channel mutations in BTX binding sites also greatly attenuate the ginsenoside Rg_3_-mediated depolarizing shift in the activation voltage observed in wild-type channels. Moreover, ginsenoside Rg_3_-mediated use-dependent inhibition was almost abolished in these mutant channels. BTX binding sites in brain- and muscle-type Na^+^ channels play important roles in ginsenoside Rg_3_-mediated Na^+^ channel interaction at cellular and molecular levels (Lee et al., 2008b).

## Ginsenoside pharmacology in the interaction with membrane receptor ligand-gated ion channels

### Regulation of GABA_A_ and glycine receptor channel activity by ginsenosides

Ginseng has been shown to have an anxiolytic-like effect in animal model studies (Kim et al., 2009). Recent studies have shown that ginsenosides interact with the GABA_A_ receptor, and may regulate the binding of the ligand with the GABA_A_ receptor. Ginsenosides differentially regulate the binding of [^3^H]-flunitrazepam or [^3^H]-muscimol to the GABA_A_ receptor in a rat brain membrane fraction (Kimura et al., 1994). On the other hand, prolonged infusion with ginsenoside Rc in the rat brain elevates [^3^H]-muscimol binding to the GABA_A_ receptor in a brain region-specific manner, while ginsenoside Rg_1_ has no effect (Kim et al., 2001). Thus, ginsenosides may regulate the GABA_A_ receptor by affecting the binding affinities of its ligands.

In a GABA_A_ receptor channel activity study, ginsenosides were also shown to regulate GABA_A_ receptor channel activity by enhancing GABA-mediated channel activity (Choi et al., 2003a). Thus, in studies using *Xenopus* oocytes expressing human recombinant GABA_A_ receptor, ginsenosides Rb_1_, Rb_2_, Rc, Rd, Re, Rf, Rg_1_, and Rg_2_ affected GABA_A_ receptor channel activity, and ginsenoside Rc most potently enhanced the GABA-induced inward peak current (*I*_GABA_). Bicuculline, a GABA_A_ receptor antagonist, and picrotoxin, a GABA_A_ channel blocker, blocked the stimulatory effect of ginsenoside Rc on *I*_GABA_. Niflumic acid (NFA) and 4,4′-diisothiocyanostilbene-2,2′-disulfonic acid, both Cl^−^ channel blockers, attenuated the effect of ginsenoside Rc on GABA-induced inward peak current.

Compared to the GABA_A_ receptor, few investigations have been carried out into the ginsenoside-mediated action on glycine receptor channel activity. In a study using human glycine α1 receptor channel expressed in *Xenopus* oocytes, Noh et al. (2003) demonstrated that although treatment with ginsenoside Rf enhanced the glycine-induced inward peak current in a dose-dependent and reversible manner, ginsenoside Rf itself did not elicit membrane currents. The effect of ginsenoside Rf action on glycine receptor channel activity was blocked by strychnine, a glycine receptor antagonist, and 4,4′-disothiocyanostilbene-2,2′-disulfonic acid (DIDS), a Cl^−^channel blocker. The various ginsenosides, listed in order of potency for the enhancement of the glycine-induced inward Cl^−^ current, were ginsenoside Rb_1_ > >Rb_2_ > Rg_2_ = Rc > Rf > Rg_1_ > Re. Further study will be helpful for elucidation of the interaction of ginsenosides with glycine receptor proteins to enhance the glycine-induced inward Cl^−^ current.

### Ginsenoside Rg_3_ interacts with the γ_2_ subunit to regulate GABA_A_ receptor channel activity

Two characteristics of ginsenoside Rg_3_-induced GABA_A_ receptor interaction have been described. First, ginsenoside Rg_3_ itself evoked an inward current in a concentration-dependent manner in *Xenopus* oocytes expressing human recombinant GABA_A_ receptor (α_1_β_1_γ_2S_) in the absence of GABA. Ginsenoside Rg_3_-elicited inward currents were blocked by a GABA_A_ receptor antagonist, indicating that ginsenoside Rg_3_ itself activates GABA_A_ receptors (Lee et al., 2013b). Ginsenoside Rg_3_-elicited inward currents were not observed in the absence of the γ_2S_ or γ_2L_ subunits. The magnitude of the ginsenoside Rg_3_-elicited inward current was dependent on the expression ratio of the γ_2S_ subunit. However, ginsenoside Rg_3_ exerted no effects in oocytes expressing other subunits, such as γ_1_, γ_3_, δ, and ε, with α_1_β_1_.

The γ_2_ subunit of the GABA_A_ receptor plays an important role in the action of human epilepsy (Bowser et al., 2002). The γ_2_ subunit of the GABA_A_ receptor is also essential for the formation of high-affinity benzodiazepine-binding sites, and mutations in benzodiazepine-binding sites greatly attenuated the benzodiazepine-induced potentiation of *I*_GABA_ (Buhr et al., 1997). In addition, increased expression of the γ_2_ subunit compared to other subunits further potentiated benzodiazepine-induced *I*_GABA_ (Boileau et al., 2003). However, GABA_A_ receptors with mutant γ_2_ subunit benzodiazepine-binding sites did not affect the action of ginsenoside Rg_3_ (Lee et al., 2013b). Thus, it is unlikely that the ginsenoside Rg_3_-induced activation of α_1_β_1_γ_2_ GABA receptors is achieved through interaction with benzodiazepine-binding sites.

Second, co-treatment of ginsenoside Rg_3_ with GABA further potentiated *I*_GABA_ in oocytes expressing GABA_A_ receptor (α_1_β_1_γ_2S_) (Lee et al., 2013b). However, the potentiating effect of ginsenoside Rg_3_ on *I*_GABA_ was not specific to the γ_2_ subunit, and co-expression of other subunits such as γ_1_, γ_3_, δ, and ε also enhanced *I*_GABA_. The degree of potentiation of *I*_GABA_ by ginsenoside Rg_3_ was not significantly different in the presence of different subunits. Even ginsenoside Rg_3_ enhanced *I*_GABA_ in oocytes expressing α_1_β_1_ subunits and ginsenoside Rg_3_ itself did not elicit inward currents in oocytes expressing α_1_β_1_ GABA_A_ receptor in the absence of GABA. Thus, ginsenoside Rg_3_ may have dual binding sites for GABA_A_ receptor modifications, one for GABA binding sites in the presence of GABA and the other one for γ_2_ subunit in the absence of GABA. However, ginsenoside Rg_3_ had no effects on GABAc receptor channel activity (Lee et al., 2013b).

### Regulation of 5-HT_3_ receptor channel activity by ginsenosides

Ginsenoside Rg_2_ and ginsenoside metabolites inhibit 5-HT_3_ receptor-mediated ion currents (*I*_5−*HT*_) in *Xenopus* oocytes expressing 5-HT_3_ receptors (Choi et al., 2003b; Lee et al., 2004a). The inhibitory effect of ginsenoside Rg_2_ on 5-HT-induced inward currents was non-competitive and voltage-independent, similar to the ginsenoside-induced modulation of heteromeric nicotinic acetylcholine receptor. In addition, the inhibitory effect of ginsenoside Rg_3_ on serotonin-induced currents (*I*_5−*HT*_) is observed when applied extracellularly but not intracellularly (Lee et al., 2004b), indicating that ginsenoside Rg_3_ regulates 5-HT_3_ receptors outside the cell.

### Ginsenoside Rg_3_ regulates 5-HT_3_ receptor channel activity through interaction with amino acids at the channel gating region

**Figure 4:**
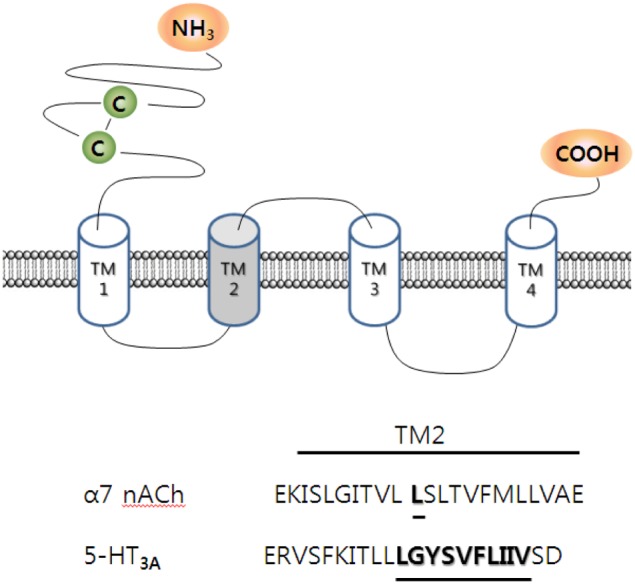
**Topology and sequence alignment of α7 nicotinic acetylcholine (nACh) and 5-HT_3A_ receptors at transmembrane domain 2 (TM2).** α7 nACh and 5-HT_3A_ receptors consist homomeric pentamer. TM2 of each segment forms a channel pore. The underlined specific amino acid residues in those ligand-gated channels are thought to interact with 20(*S*)-ginsenoside Rg_3_ (Lee et al., 2007, 2009).

Mutations in V291A, F292A, and I295A in the transmembrane domain 2 (TM2) greatly attenuated or abolished ginsenoside Rg_3_-induced inhibition of peak *I*_5−*HT*_. Thus, ginsenoside Rg_3_ acts through the 5-HT_3_ receptor protein, and alterations in TM2 of the 5-HT_3_ receptor could affect the action of ginsenoside Rg_3_ (Lee et al., 2007). Interestingly, the V291A mutation, although not the F292A or I295A mutations, induced constitutively active ion currents, with a decreased current decay rate. Ginsenoside Rg_3_ treatment of this mutant receptor accelerated the rate of current decay in the presence of 5-HT, suggesting that the presence of ginsenoside Rg_3_ caused channel closure rather than opening. Thus, ginsenoside Rg_3_ and TMB-8, an open channel blocker, inhibited the constitutively active ion currents. Diltiazem, another open channel blocker, did not prevent ginsenoside Rg_3_-induced inhibition of the constitutively active ion currents in occlusion experiments (Lee et al., 2007). This report provides the following insight: first, ginsenoside Rg_3_ inhibits 5-HT_3A_ receptor channel activity through interaction with residues V291, F292, and I295 in the channel gating region of TM2, and second, ginsenoside Rg_3_ regulates 5-HT_3A_ receptor channel activity in the open state at different site(s) from those used by TMB-8 and diltiazem. Thus, ginsenoside Rg_3_ inhibits the 5-HT_3_ receptor in the open state through interactions with V291, F292, and I295. The identification of ginsenoside Rg_3_ interacting sites in the 5-HT_3_ receptor indicates that ginsenoside Rg_3_ achieves its pharmacological actions via a specific interaction with the 5-HT_3_ receptor (Figure 4).

### Regulation of nicotinic acetylcholine receptor channel activity by ginsenosides

Ginsenosides inhibit acetylcholine-stimulated catecholamine release from chromaffin cells, which mainly contain α3β4 nicotinic acetylcholine receptors involved in catecholamine release (Tachikawa et al., 2001). Furthermore, ginsenosides also inhibited acetylcholine-induced inward currents in oocytes expressing nicotinic receptor α1β1δε or α3β4 subunits, suggesting that ginsenosides directly regulate nicotinic acetylcholine receptor channel activities (Choi et al., 2002). However, ginsenosides themselves had no effect on basal currents in oocytes expressing nicotinic acetylcholine receptor αβδε or α3β4 subunits. The inhibition of acetylcholine-induced inward currents by ginsenosides in oocytes expressing nicotinic acetylcholine receptor αβδε or α3β4 subunits was reversible, voltage-independent, and non-competitive, indicating that ginsenosides may bind to the nicotinic acetylcholine receptor when channels are open, but do not compete with acetylcholine to regulate these receptors (Choi et al., 2002). Interestingly, it appears that protopanaxatriol ginsenosides such as Re, Rf, Rg_1_, or Rg_2_ were more potent than protopanaxadiol ginsenosides such as Rb_1_, Rb_2_, Rc, and Rd in inhibiting acetylcholine-induced inward currents (Choi et al., 2002). On the other hand, ginsenoside Rg_2_ reduced the peak current and increased the desensitization of acetylcholine-induced inward currents in oocytes expressing human neuronal nicotinic acetylcholine receptors such as α3β4, α3β2, α4β4, and α4β2 (Sala et al., 2002). Thus, the inhibitory effects of ginsenosides on acetylcholine-stimulated attenuation of catecholamine release are achieved through nicotinic acetylcholine receptor channel activity.

In *Xenopus* oocytes heterologously expressing the α9α10 nicotinic acetylcholine receptor, ginsenosides blocked acetylcholine-induced inward currents (*I*_*ACh*_), listed in order of potency, with ginsenoside Rg_3_ > Rb2 > CK > Re = Rg2 > Rf > Rc > Rb1 > Rg1 in a reversible manner, and the blocking effect of ginsenoside Rg_3_ on *I*_*ACh*_ was the same after pre-application compared to co-application of ginsenoside Rg_3_. Ginsenoside Rg_3_-induced *I*_*ACh*_ inhibition was not affected by acetylcholine concentration and was independent of membrane holding potential. The inhibitory effect of ginsenoside Rg_3_ on *I*_*ACh*_ was not observed in oocytes expressing the α9 subunit alone, indicating that the presence of the α10 subunit is required for ginsenoside Rg_3_-induced regulation of α9α10 nicotinic acetylcholine receptor channel activity (Lee et al., 2013a). The α10 subunit of the α9α10 nicotinic acetylcholine receptor may play an important role in ginsenoside Rg_3_-induced interaction with the α9α10 nicotinic acetylcholine receptor.

### Identification of ginsenoside interaction sites in the regulation of the nicotinic acetylcholine receptor

In contrast to heteromeric acetylcholine receptors, ginsenosides had no effect on wild-type homomeric α7 nicotinic acetylcholine receptor-mediated ion currents (Lee et al., 2009). The homomeric α7 nicotinic acetylcholine receptors, which are predominantly expressed in the cortical and limbic areas, are the major binding sites for α-bungarotoxin in the mammalian central nervous system and are Ca^2+^ permeable (Gotti et al., 2009). The α7 nicotinic acetylcholine receptor and the 5-HT_3A_ receptors are both homomeric ligand-gated ion channels. Interestingly, single point mutations of Leu247 to Thr247 in the highly conserved TM2, which forms the channel pore region, creates gain-of-function alterations, including slower desensitization, increased acetylcholine affinity, and a linear current-voltage relationship, as well as altering pharmacological properties such as the conversion of various α7 nicotinic acetylcholine receptor antagonists into agonists (Revah et al., 1991; Bertrand et al., 1992). Thus, the L247 residue of the α7 nicotinic acetylcholine receptor could be a useful target for drug development and a focal point in the investigation of channel gating of the acetylcholine receptor (Palma et al., 2006). In addition, the Leu residue corresponding to position 247 of the chick α7 nicotinic acetylcholine receptor channel is highly conserved in all nicotinic, GABA_A_, 5-HT_3_, and glycine receptors, and is believed to be positioned at the gate (Lester et al., 2004). Moreover, recently acquired high resolution structures of the nicotinic acetylcholine receptor channel show that the conserved Leu is located at the narrowest part of the channel, and that the side chain of the amino acid head points toward the lumen of the pore (Miyazawa et al., 2003). The importance of position 247 for gating and conductance has also been demonstrated in a functional study (Bertrand et al., 1992).

The mutation of Leu247 to various other amino acid residues induces changes in the receptor sensitivity to ginsenoside Rg_3_. Interestingly, mutations in L247 to L247A, L247D, L247E, L247I, L247S, and L247T, although not L247K, rendered mutant receptors sensitive to ginsenoside Rg_3_ (Lee et al., 2009). Ginsenoside Rg_3_ inhibition of the mutant α7 nicotinic acetylcholine receptor channel currents was reversible and concentration-dependent. In addition, ginsenoside Rg_3_ inhibition of the mutant α7 nicotinic acetylcholine receptor was strongly voltage-dependent and non-competitive. The homology docking model between ginsenoside Rg_3_ and the mutant receptor revealed that ginsenoside Rg_3_ forms hydrogen bonds with amino acids, such as Ser240 of subunit I and Thr244 of subunits II and V at the channel pore, whereas the wild-type receptor ginsenoside Rg_3_ localizes at the interface of the two wild-type receptor subunits. Thus, the mutation of Leu247 to Thr247 may induce conformational changes in the wild-type receptor, creating a binding pocket for ginsenoside Rg_3_ at the channel pore (Figure 4).

### Regulation of N-methyl-D-aspartate (NMDA) receptor channel activity by ginsenosides

Ginsenosides Rb_1_ and Rg_3_ have been shown to attenuate glutamate- and NMDA-induced neurotoxicity by inhibiting the overproduction of nitric oxide, the formation of malondialdehyde, and the influx of Ca^2+^ in rat cortical cultures (Kim et al., 1998b). In addition, in rat hippocampal cultures, ginsenosides and ginsenoside Rg_3_ attenuated high K^+^-, glutamate-, and NMDA-induced Ca^2+^ influx (Kim et al., 2002). Seong et al. (1995) showed that ginsenosides attenuated glutamate-induced swelling of cultured rat astrocytes. Notoginsenoside R1 prevents glutamate-mediated neurotoxicity in cultured mouse cortical neurons (Gu et al., 2009). On the other hand, in an *in vivo* study using anesthetized rats, intracerebroventricular administration of ginsenoside Rb_1_, and not Rg_1_,significantly inhibited the magnitude of long term potentiation induced by strong tetanus in the dentate gyrus, although ginsenoside Rb_1_ did not affect the basal synaptic responses evoked by low-frequency tests (Abe et al., 1994). Pretreatment with ginsenosides via the intrathecal route attenuated NMDA- or substance P-induced nociceptive behavior, but had no effect on glutamate-induced behavior (Yoon et al., 1998; Shin et al., 1999). Pretreatment of ginsenosides via the intraperitoneal route also attenuated cell death of hippocampal neurons induced by kainate (Lee et al., 2002). Ginsenosides Rh2 and Rg_3_ inhibit NMDA receptor channel currents in cultured rat hippocampal neurons (Lee et al., 2006a). Regarding the effects of ginsenoside metabolites such as compound K (CK), protopanaxadiol (PPD), and protopanaxatriol (PPT) on NMDA receptor channel activity, PPT, unlike CK and PPD, reversibly inhibited NMDA-mediated inward currents (*I*_*NMDA*_) in a concentration-dependent manner. PPT inhibition of *I*_*NMDA*_ was non-competitive with NMDA, and was independent of the membrane holding potential (Shin et al., 2012), even though ginsenoside Rh2, Rg_3_, and PPT interact with the NMDA receptor.

### Identification of ginsenoside interaction sites in the regulation of the NMDA receptor

Ginsenoside Rg_3_ does not compete with NMDA in NMDA receptor-mediated ion current inhibition in rat hippocampal neuron cultures (Kim et al., 2004b; Lee et al., 2006a). In addition, ginsenoside Rg_3_ does not interact with Mg^2+^ or phencyclidine (PCP, MK-801, ketamine) binding sites. However, the inhibitory effect of ginsenoside Rg_3_ on NMDA receptor-mediated ion currents was attenuated by increasing glycine concentrations, whereas ginsenoside Rh2-mediated inhibition of NMDA receptor-mediated ion currents were attenuated by increasing spermine concentrations. Thus, ginsenoside Rg_3_ and ginsenoside Rh2 selectively targeted NMDA receptors with different NMDA receptor regulatory sites (Lee et al., 2006a). These reports show the possibility that ginsenoside Rg_3_ is a competitive antagonist at the glycine- and polyamine-binding site, although the specific amino acid(s) involved in ginsenoside Rg_3_ binding have not been identified. Site-directed mutagenesis may provide further information about the interaction of ginsenoside Rg_3_ with glycine binding site(s).

## Chacterizations of ginsenoside Rg_3_-mediated actons on ion channels and receptors

Ginsenosides exhibit several principles in their actions of a variety of ion channels and receptors. Firstly, ginsenosides do not affect most of ion channel and receptor activities, when they are at rest or without ligand stimulations (Nah et al., 2007). But, when voltage-gated ion channels are depolarized or receptors are activated by the relevant receptor agonists, ginsenosides affect ion channel or ligand-gated ion channel activities. Thus, conformational changes to ion channels or receptor proteins induced by depolarization or receptor ligand binding may provide an opportunity for ginsenoside Rg_3_ to bind ion channels or receptors to exert its action (i.e., Na^+^ channels and 5-HT_3_ receptor) (Lee et al., 2007, 2008c).

Secondly, in ginsenoside Rg_3_-induced voltage-gated ion channel regulations, the ginsenoside Rg_3_ effects on voltage-gated Ca^2+^, K^+^, and Na^+^ channel currents were achieved via interaction with transmembrane domain-I-segment 6, which is known to form a channel pore or pore entryway. Ginsenoside Rg_3_-induced inhibition of L-type Ca^2+^ channel currents was attenuated by mutations of Leu427, Asn428, and Leu431 in transmembrane domain-I-segment 6 residues (Choi et al., 2009). Similarly, N418K and L421K mutant rat brain Na_*V*_1.2, and L437K mutant mouse skeletal muscle Na_*V*_1.4 channel attenuated or abolished ginsenoside Rg_3_ inhibition of Na^+^ currents. Another interesting observation was the consistent pattern exhibited in ginsenoside Rg_3_-mediated action on various K^+^ channel subtypes. As shown in Figure 3, K^+^ channels have a common feature, in that they all have a pore-lining P-loop with a consensus amino acid sequence –TXGYGD–, which is called the K^+^ channel “signature sequence” (Heginbotham et al., 1994). These residues, repeated in each of the 4 α subunits, form the K^+^ selectivity filter. Ginsenoside Rg_3_-mediated human Kv1.4, KCNQ (*I*_*Ks*_), hERG, and BK_Ca_ K^+^ channels are regulated through interaction with K531 and K318, V319, S631, and Y360 residues respectively, all of which are the first or second amino acid after –TXGYGD–. They are all located at the channel pore entry (Lee et al., 2008a; Choi et al., 2010, 2011a,b) (Figure 2). Thus, ginsenoside Rg_3_ has a common interaction site near the “signature sequence” in the subsets of the K^+^ channels examined. However, the interaction of ginsenoside Rg_3_ with these K^+^ channel subtypes exhibits differential effects (i.e., activation of BK_Ca_, *I*_*Kr*_, *I*_*Ks*_ or inhibition of Kv1.2 and Kv4.2). Another important characteristic in Kv1.4 and BK_Ca_ channel interaction with ginsenoside Rg_3_ is the sharing of other K^+^ channel regulator binding sites such as the extracellular TEA binding site, although TEA structure is different from that of ginsenoside (Lee et al., 2008a).

Thirdly, ginsenoside Rg_3_ exhibits its effects via differential interactions with ligand-gated ion channels. Ginsenoside Rg_3_-induced actions of 5-HT_3_ and mutant α7 nicotinic acetylcholine receptor are achieved via interaction with amino acids in TM2, which forms channel pore (Choi et al., 2009). Ginsenoside Rg_3_ regulates GABA_A_ receptor channel activity via γ_2_ subunit, whereas ginsenoside Rg_3_ regulate NMDA receptor channel activity via allosteric interaction sites such as co-agonist, glycine or polyamines, binding site.

Finally, there might be a compensatory action by ginsenoside action in ion channel regulations. For example, ginsenoside Rg_3_ stabilizes membrane potentials through the inhibition of both Ca^2+^ and Na^+^ channels, but ginsenoside Rg_3_ might induce a depolarization of neuronal cells by inhibiting Kv1.4 and Kv4.2 channel activity. Thus, ginsenoside Rg_3_ action is interesting because the same inhibitory effects on Ca^2+^, Kv1.4 and Kv4.2, and Na^+^ channels could result in opposite effects in neurons. One possible speculation on the actions of ginsenoside Rg_3_ on nervous system is that suppression of neuronal excitability by ginsenoside Rg_3_-mediated Ca^2+^ and Na^+^ channel inhibition could be to some extent compensated by inhibition of Kv1.4 and Kv4.2 channel activity. On the other hand, there is no evidence that ginsenoside directly binds to ion channels or receptors, since most of studies are indirectly obtained from mutation experiments. In future studies, it will be needed to obtain direct evidence(s) to confirm ginsenoside binding to ion channel or ligand-gated ion channel proteins.

## A linkage of the ginsenoside-mediated ion channels and receptor interactions between ginseng pharmacology

**Figure 5:**
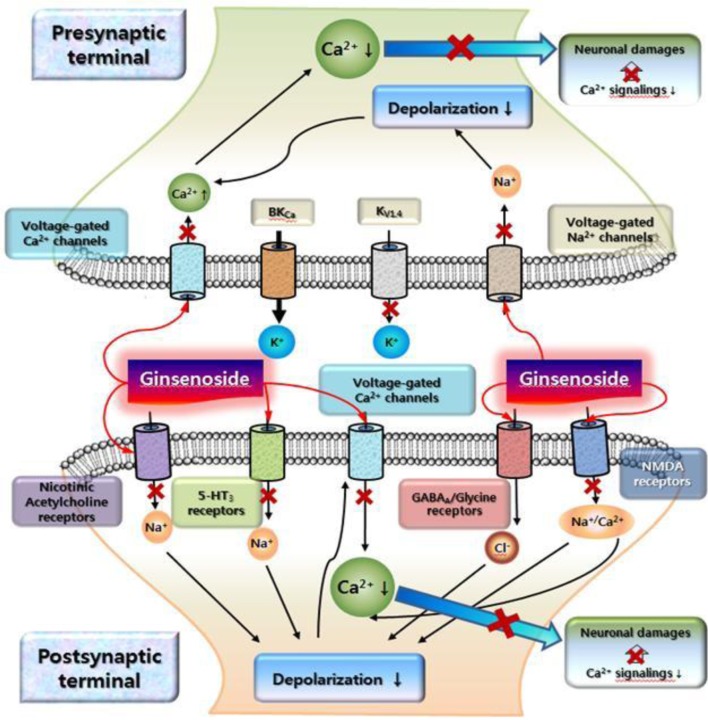
**A schematic illustration on cytosolic Ca^2+^ overload signalings and ginsenoside-mediated attenuation against cytosolic Ca^2+^ overload.** This schematic drawing shows that cytosolic Ca^2+^ levels could be elevated in ischemic or traumatic brain injury. The elevation of cytosolic Ca^2+^ levels occurs either via direct activation of voltage-gated Ca^2+^ channels or via depolarization caused by voltage-dependent Na^+^ channel activation and other excitatory ligand-gated ion channels at pre- and post-synaptic sites. The overload of cytosolic Ca^2+^ levels caused by excitatory neurotransmitters or neurotoxins may initiate the persistent activation of Ca^2+^-dependent signaling, resulting in damage, for instance through apoptosis or necrosis. Although ginsenoside has no effects on ion channels and ligand-gated ion channels at rest state of neurons, ginsenoside might exhibit its effects by attenuation of cytosolic Ca^2+^ elevation by abnormal conditions by inhibiting ion channels and receptors (as indicated with “x” in arrow). The detailed explanations were described in text. Ginsenoside exhibits differential regulations on subsets of K^+^ channels. So, one possible hypothesis is that K^+^ channels regulated by ginsenoside might play a balancing role on Ca^2+^ and Na^+^ channel inhibitions by ginsenoside.

In traditional medicine, ginseng was utilized as a tonic to invigorate body (Nah et al., 2007). Ginsenoside-mediated actions of ion channels and receptors could underlie molecular bases for the explanations of traditional ginseng pharmacology. Here, this review illustrates representative examples how ginsenoside-mediated actions of ion channels and receptors link to ginseng pharmacology. In normal Ca^2+^ homeostatic state, cytosolic calcium plays a key role for learning, memory and other many cellular events (Berridge et al., 1998). In contrast, Ca^2+^ dyshomeostasis is one of important events in brain ischemic and traumatic brain injury (Wojda et al., 2008). Ginsenoside might be helpful for neuroprotection by negative coupling of [Ca^2+^]_i_ in Ca^2+^ dyshomeostasis of nervous systems. Brain injury will cause ATP exhaustion by mitochondrial dysfunction and also subsequently couples to the slow secondary excitotoxicity by glutamate (Pang and Geddes, 1997). The secondary glutamate-induced excitotoxicity in ATP deficient neurons is initiated by voltage-dependent Na^+^ channel activation, which is coupled to membrane depolarization, Ca^2+^ channel activation, and subsequent NMDA receptor activation (Alzheimer, 2002). Moreover, pathologically elevated Na^+^ and Ca^2+^ levels in the cytosol are likely to trigger a cascade of molecular events that eventually lead to neuronal death (e.g., formation of reactive oxygen species, lipid peroxidation, mitochondrial dysfunction, activation of calpain and caspases, etc.). As described above, ginsenosides inhibit cytosolic Ca^2+^ and Na^+^ elevation through these channel activations as well as NMDA receptor inhibition (Lee et al., 2006a). Thus, negative couplings of ginsenosides to Ca^2+^ and Na^+^ channels and NMDA receptors might be helpful for the attenuation of excitotoxity under the brain ischemic and traumatic brain injury. Figure 5 illustrate a schematic example based on reports how ginsenoside-mediated interactions with Ca^2+^ and Na^+^ channels and NMDA receptors may be coupled to neuroprotective effects against Ca^2+^ dyshomeostasis induced by uncontrolled Ca^2+^ and Na^+^ channels or NMDA receptors (Lee et al., 2002, 2006a; Kim and Rhim, 2004; Park et al., 2004; Kim et al., 2005c; Lian et al., 2005; Tian et al., 2005; Kim et al., 2007, 2008b; Zhang et al., 2008; Cheon et al., 2013).

Ginseng and ginsenosides have also been reported to alleviate stress-induced symptoms and lesions (Attele et al., 1999). Stress is one of main causes for development of almost all types of diseases. The adrenal gland is one of the peripheral organs responding to stress. In stress situation, the adrenal gland secrets catecholamines from the medulla and renders the organs to cope with stress. α3β4 nicotinic acetylcholine receptors in adrenal medulla play a key role in catecholamine secretion. Ginsenosides and their metabolites inhibit α3β4 nicotinic acetylcholine receptor channel activity and catecholamine release (Tachikawa et al., 2001; Choi et al., 2002). Thus, ginsenosides might be helpful for alleviation of stress by controlling catecholamine secretion during over-stress situation.

On the other hand, traditional medicine also showed that ginseng is utilized for the alleviation of emesis, which includes nausea and vomiting. Nausea and vomiting are significant adverse effects of anti-cancer agents such as cisplatin, and cause significant patient morbidity. 5-HT_3_ receptors are involved in vomiting and irritable bowel syndrome (Kim et al., 2005a,b). Ginsenoside-mediated action of 5-HT_3A_ receptor channel activity may be the molecular basis of the anti-vomiting action of ginseng. Ginsenoside might be used for attenuation of anticancer agent-induced side effects of vomiting and nausea.

There are multiple cardiovascular effects attributed to ginseng, including cardioprotection, antihypertensive effects, and attenuation of myocardial hypertrophy, heart failure, and the ischemic and reperfused heart in a variety of experimental models (Attele et al., 1999). Ginseng total saponins and ginsenoside Rg_3_ regulate L-type Ca^2+^ and various K^+^ channel activities. Especially, ginsenoside Re enhances *I*_*Kr*_ and *I*_*Ks*_ channel currents in guinea myocytes (Bai et al., 2003, 2004). Ginsenoside Rg_3_ also delays deactivation of *I*_*Ks*_ and enhances *I*_*Kr*_. In heart, *I*_*Kr*_ and *I*_*Ks*_ channels are clinically important and are target for drug development, since dysfunction of these channels is associated with sudden death in human (Bai et al., 2004). Thus, ginsenosides-mediated actions of *I*_*Kr*_ and *I*_*Ks*_ channels as well as K^+^ channels might contribute to cardioprotective effects of ginseng.

## Summary

The pharmacological behaviors of ginseng are diverse rather than unique, since ginsenosides, as one of active ingredients of ginseng, exhibit differential pharmacological actions in ion channels or ligand-gated ion channel regulations. Therefore, it is unlikely that ginseng ginsenoside achieves its diverse effects on ion channels and receptors via mediation of its own receptor activations on plasma membrane for the following reasons. Firstly, ginsenoside affinity for ion channels and receptors is very low compared to other receptor-specific ligands or toxins (Table 1). Rather, ginsenosides directly interact with various ion channel proteins and receptors without selectivity (Table 1). Secondly, since ginsenoside does not have its receptor on plasma membrane, ginsenoside does not induce spontaneous cellular responses in ion channel or receptor activity without stimuli such as depolarization or receptor ligands. Thus, diverse actions of ginsenoside on ion channels and receptors without its specific receptor mediations could be a limitation on its therapeutic use. Nevertheless, since ginseng influences various body functions as a traditional medicine, a pharmacological role of ginsenosides through the interactions with diverse ion channels and receptors might have potential as a prevention or treatment for a variety of nervous system disorders.

**Table 1:** **Summary of EC_50_ or IC_50_ on ginsenoside-induced inhibition or stimulation of various voltage-gated ion or ligand-gated ion channel activities**.

**Voltage-gated ion channels**	**Ginsenoside (Effect)**	**EC_50_ or IC_50_ (μM)**	**Interaction sites**	**References**
**Ca^2+^ CHANNELS IN CULTURED CELLS**				
Sensory neurons	Rf (Inhibition)	40		Nah et al., 1995
Chromaffin cells	GTS (Inhibition)	ND		Choi et al., 2001
Sensory neurons	GTS and Rg_3_ (Inhibition)	ND		Rhim et al., 2002
Hippocampal neurons	Rb1 (Inhibition)	ND		Lin et al., 2012
Cardiomyocytes	Re (Inhibition)	ND		Bai et al., 2004
**Ca^2+^ CHANNEL SUBTYPES EXPRESSED IN *XENOPUS* OOCYTES**				
L	Rg_3_ (Inhibition)	39.9 ± 9.5	L417, N428, L431	Lee et al., 2006b
N	Rg_3_ (Inhibition)	64.4 ± 13.6		Lee et al., 2006b
P/Q	Rg_3_ (Inhibition)	29.6 ± 11.3		Lee et al., 2006b
R	Rg_3_ (Inhibition)	57.5 ± 12.5		Lee et al., 2006b
T	Rg_3_ (Inhibition)	97.3 ± 12.4		Lee et al., 2006b
**K^+^ CHANNELS IN CULTURED CELLS**				
K_Ca_ in vascular smooth muscle	GTS (Activation)	ND		Li et al., 2001
*I*_*Ks*_ in cardiomyocytes	Re (Activation)	1.4 ± 0.4		Furukawa et al., 2006
**K^+^ CHANNELS EXPRESSED IN *XENOPUS* OOCYTES**				
Kv1.4	Rg_3_ (Inhibition)	32.6 ± 2.2	K531	Lee et al., 2008a
BK_Ca_	Rg_3_ (Activation)	15.3 ± 3.1	Y360	Choi et al., 2011a
hERG (*I*_*Kr*_)	Rg_3_ (Activation)	0.41 ± 0.05	S631	Choi et al., 2011b
KCNQ (*I*_*Ks*_)	Rg_3_ (Activation)	15.2 ± 8.7	K318, V318	Choi et al., 2010
**Na^+^ CHANNELS EXPRESSED IN tsA201 CELLS**				
Brain_2*a*_ Na^+^ channel	GTS (Inhibition)	ND		Liu et al., 2001
**Na^+^ CHANNELS EXPRESSED IN *XENOPUS* OOCYTES**				
Nav1.2	Rg_3_ (Inhibition)	32.0 ± 6.0	I417, N418, L421	Lee et al., 2008b
Nav1.4	Rg_3_ (Inhibition)	58.5 ± 6.3	I433, N434, L437	Lee et al., 2008b
Nav1.5	Rg_3_ (Inhibition)	16.1 ± 2.8		Kang et al., 2005
**LIGAND-GATED ION CHANNELS EXPRESSED IN *XENOPUS* OOCYTES**				
GABA_A_	Rc (Activation)	53.0 ± 12.3		Choi et al., 2003a,b
Glycine	Rf (Activation)	49.8 ± 9.8		Noh et al., 2003
5-HT_3_	Rg_3_ (Inhibition)	27.6 ± 4.3	V291, F292, I295	Lee et al., 2007
Nicotinic acetylcholine				
α3β4	Rg_2_ (Inhibition)	60 ± 14		Choi et al., 2002
α1β1δε	Rg_2_ (Inhibition)	16 ± 9		Choi et al., 2002
α7 (L247A mutant)	Rg_3_ (Inhibition)	33.1 ± 1.3	L247	Lee et al., 2009
NMDA	Protopanaxatriol (Inhibition)	48.1 ± 16		Shin et al., 2012

EC_50_ (BK_Ca_, hERG, and KCNQ (I_Ks_) K^+^ channels, and GABA_A_ and glycine receptors) or IC_50_ (the remainder) values were determined in oocytes expressing the specified ion channels or receptors. GTS, ginseng total saponin fraction; ND, not determined.

### Conflict of interest statement

The author declares that the research was conducted in the absence of any commercial or financial relationships that could be construed as a potential conflict of interest.
